# Comparison of Radiography and Computed Tomography for Evaluation of Third Carpal Bone Fractures in Horses

**DOI:** 10.3390/ani13091459

**Published:** 2023-04-25

**Authors:** Catherine Steel, Benjamin Ahern, Steven Zedler, Stuart Vallance, Lawrence Galuppo, Jennifer Richardson, Christopher Whitton, Alex Young

**Affiliations:** 1 Department of Veterinary Clinical Services, The Hong Kong Jockey Club, Sha Tin, New Territories, Hong Kong; 2 Equine Specialist Hospital, The University of Queensland, Gatton, QLD 4343, Australia; 3 Advantage Equine, Ascot Vale, VIC 3032, Australia; 4 Surgical and Radiological Sciences, School of Veterinary Medicine, University of California, Davis, CA 95616, USA; 5 Murdoch Veterinary School, Murdoch University, Murdoch, WA 6150, Australia; 6 Werribee Equine Centre, 250 Princes Hwy, Werribee, VIC 3030, Australia

**Keywords:** horse, radiography, computed tomography, carpus, slab fracture

## Abstract

**Simple Summary:**

The extent of injury in horses with third carpal bone (C3) fracture is not fully appreciated using conventional radiography. Computed tomography (CT) enables more detailed imaging of bone needed for accurate diagnosis and is becoming more widely available in equine practice. We describe the CT findings in racehorses with a variety of C3 fracture configurations (15 cadaver carpi and 27 carpi from 26 horses with C3 fracture) and report agreement with radiographic findings in those with ante-mortem digital radiographs (DR). CT detected more concurrent bone pathology and enabled more accurate determination of fracture configuration. Agreement between DR and CT was good for recognition of fracture displacement, fair for fracture comminution, bone loss at the articular surface and concurrent osteochondral fragmentation, and poor to slight for recognition of whether the fracture was complete in each plane, and whether additional fissures and bone lucencies were present. This study highlights the limitations of radiography and the benefits of CT in the accurate diagnosis of bone pathologies in horses with C3 fracture.

**Abstract:**

Radiographs underestimate the extent of bone injury in horses with third carpal bone (C3) fractures (Fx). We aimed to describe bone pathologies identified using computed tomography (CT) and compare the diagnostic value of digital radiography (DR) and CT in horses with C3 Fx. CT images of 15 racehorses with C3 Fx and 10 controls were reviewed (Part 1) then DR and CT images of 26 racehorses (24 Thoroughbred, 2 Standardbred) with C3 Fx (Part 2) were evaluated. Agreement on fracture geometry and concomitant bone lesions was tested between DR and CT using the kappa statistic (Part 2). For agreement analysis, 38 limbs were used (27 Fx carpi from 26 horses and 11 contralateral carpi). Intermodality agreement was good for recognition of displacement, fair for comminution, articular surface bone loss and osseous fragmentation, and poor–slight for recognition of whether the Fx was complete, additional fissures and lucencies. CT provides more detailed information than DR regarding bone pathology and fracture configuration in horses with C3 fracture. Correlation of CT findings with clinical information and outcome needs to be explored; however, the more accurate diagnosis possible with CT is likely valuable when deciding on the most appropriate management and for surgical planning.

## 1. Introduction

Radiography plays an important role in the diagnosis of carpal injury but has well-recognized limitations. For some horses with third carpal bone (C3) fractures (Fx), the poor sensitivity of planar radiography to identify fracture configuration and the full extent of comminution and other injury has been reported [1,2,3,4,5,6,7,8]. Additional views to the standard radiographic series can be useful in some cases including those with suspected sagittal Fx of the radial facet of C3 (RaF) [9], but not all limitations can be overcome. Arthroscopy allows the identification and management of some concurrent lesions [5]; yet, not all bone lesions are evident arthroscopically. Early and accurate diagnosis in all cases would be advantageous before decisions are made concerning the best course of management and likely prognosis.

In comparison, computed tomography (CT) is a cross-sectional modality with increased contrast resolution and no superimposition and is becoming more widely available in equine referral practice. CT is extremely useful in the diagnosis of distal limb fractures in horses, and when other imaging techniques are inconclusive [7,8,10,11,12,13] and can detect fractures, comminution, small osseous fragments (OF), periosteal new bone, osteoarthritis (OA), lucencies within the subchondral cortical plate and subchondral trabecular bone (collectively termed subchondral bone, SCB) or osseous cyst-like lesions (OCLL) at various sites not evident radiographically [7,8,12,14,15,16,17,18,19,20,21,22]. Due to benefits over planar radiography, CT helps guide surgical approach in horses with a range of distal limb fractures [8,23] and determination of prognosis in horses with lateral condylar Fx [23]. The value of pre-operative CT in evaluation of carpal fractures has been reported; yet, only a limited number of horses with third carpal fracture were included [8]. Assessment of the value and limitations of radiography in evaluation of C3 Fx and a better understanding of radiographic lesions compared to CT findings will aid practitioners in the interpretation of radiographic studies.

The purpose of this study was to describe the CT findings in horses with C3 fracture and to determine the level of agreement of fracture morphology and bone lesions on planar digital radiography (DR) compared to CT. We hypothesised that the extent of bone pathology in horses with C3 Fx would be underestimated on DR, and that pathologic changes would be recognized with higher certainty with CT compared to DR.

## 2. Materials and Methods

### 2.1. Part 1 (Cadaver Study)

Paired cadaveric carpi from 15 thoroughbred (TB) race horses euthanised with C3 Fx and single or paired carpi from 10 TB horses (5 untrained and 5 trained) euthanised for unrelated reasons and no known history of carpal lameness were stored chilled at 4 °C and later examined by CT (Siemens Emotion 16 slice, Erlagen, Germany 2006). The entire limb or isolated carpus was positioned so that direct transverse and dorsal slices were acquired at 0.75 mm intervals and displayed at 1 mm.

Images were viewed in ‘digital imaging and communications in medicine’ (DICOM) format on an interactive DICOM viewer workstation (Osirix MD) by the primary investigator (C.S.). CT findings including location and appearance of vascular channels and sclerosis were recorded, maximal proximal and distal SCB thickness were measured using digital calipers on dorsal images, and other findings (including dorsal cortical lucencies (DCL), subchondral bone erosions/lucencies (SCBL), trabecular lucencies, fissures and fractures) were recorded and compared to radiographic findings, if available, and were used to develop an evaluation scheme for part 2. As all the Fx cases were collected before 2003 when DR was not accessible, only conventional film radiographs (XR) were available (exposure 63–66 kVp and 10 mAs, FFD 40 cm, high detail intensifying screens (18 × 24 cm Fuji EC-MA) and mammography film (Fuji AD mammography medium film) for ante-mortem examination, and these cases were excluded from part 2.

### 2.2. Part 2 (Animals)

Horses that had DR and CT examination for investigation of C3 Fx between July 2002 and October 2022 (including approximately a 3-year period at the Veterinary Medical Teaching Hospital, University of California, Davis, CA, USA, an 11-year period at the U-Vet Equine Centre, University of Melbourne, Werribee, Australia and a 5-year period at the UQ Vets Equine Specialist Hospital, University of Queensland, Gatton, Australia) were included if good quality digital radiographs of the carpus were available, including all standard series views. Cases with C3 Fx that (1) extended from the proximal to distal articular surfaces (‘slab’ fracture), (2) involved only the proximal articular surface (incomplete frontal or sagittal Fx), or (3) had complete Fx that involved the proximal articular surface, extended at least one third of the distance from the proximal to distal articular surface of C3 (uni-articular or ‘partial slab’ and ‘corner fracture’) were included [24,25,26].

**Radiographic Examination**

Radiographic examination was performed in standing, sedated horses before CT examination. Radiographic projections in all horses (standard series) included dorso-palmar (D-Pa), lateral-medial (L-M) weight bearing, L-M flexed, dorsolateral-palmaromedial oblique (D55°L-PaMO), dorsomedial-palmarolateral oblique (D55°M-PaLO or Pa55°L-DMO), and flexed dorsoproximal-dorsodistal oblique (Flexed D35°Pr-DDiO ‘skyline distal row’. Additional lesion oriented projections were taken in some cases, including a flexed D65°Pr-DDiO ‘skyline proximal row’, and if sagittal Fx was suspected but DR inconclusive, a DPrL-PDiMO projection with a range of obliquity from 15–35° (‘lateral oblique skyline’) [9]. Carpi were radiographed using a computed radiography system at 65–70 kV and 4–5 mAs and a focus-film distance of approximately 1 m for standing views and 40 cm for ‘skyline’ views.

**Computed Tomography Examination**

CT imaging was performed either under general anaesthesia (GA) immediately before surgery, or standing. CT machines and acquisition variables are listed in Table 1. For imaging under general anaesthesia, horses were positioned in lateral recumbency with forelimbs extended with the metacarpus parallel to the CT table. In-plane slices were acquired at 0.50–2.0 mm intervals from the level of the distal radius to the proximal metacarpus, reformatted into the transverse, dorsal and sagittal planes and displayed at 1 mm slice thicknesses with no interslice overlap. Images were obtained with a bone algorithm in all horses and soft tissue algorithm in all but five horses. The window and level settings were adjusted arbitrarily by each of the reviewers.

**Image Analysis**

Images were viewed independently in ‘digital imaging and communications in medicine’ (DICOM) format on an interactive DICOM viewer workstation (Osirix MD) by 2 investigators, an equine surgeon (FANZCVS) and Diplomate ACVSMR (C.S.) and a specialist in veterinary equine diagnostic imaging (ACVR-EDI) (A.Y.) who had agreed on the evaluation scheme that included 62 parameters (Supplementary Table S1) based on clinical experience and findings from Part 1. DR images were evaluated without knowledge of CT findings, and CT images were then evaluated. The observers recorded whether DR images were of acceptable quality and included minimum views. Predefined criteria as well as case data (breed, age, sex, limb) were recorded in an Excel file. Observers were also given the option of ‘unsure’ or ‘unable to assess’. Any discrepancies in interpretation of findings between observers within each imaging modality were then discussed, images reviewed and a consensus reached.

Parameters recorded included Fx plane (frontal, sagittal, other), location (radial facet (RaF), intermediate facet (InF), or both facets) and extent (incomplete or complete). Presence of comminution was recorded using a semi-quantitative grading system as absent (0, simple Fx), mild, moderate or severe and any fissure lines described. Displacement was graded as absent (0), mild (<5 mm), moderate (5–10 mm) or severe (>10 mm). Bone loss (a ‘trough’) at the proximal articular surface was described as absent, small (<5 mm), medium (5–10 mm) or large (>10 mm) and fragmentation within the Fx noted. Observers also recorded the presence of subchondral bone lysis (SCBL), dorsal cortical lucencies (DCL), osteolytic lesions within the trabecular bone or osseous cyst-like lesions (OCLL), osseous fragments (OF), bone debris in the palmar aspect of the joint, dorsal cortical modelling, entheseous new bone or other abnormalities including lesions affecting other carpal bones and the distal radius for both modalities.

Sclerosis was defined as increased bone mineral opacity on DR and as increased bone attenuation on CT. For DR, sclerosis was subjectively graded very mild, mild, moderate or severe on Pr-DDiO projections [27]. It was assessed on CT in the transverse, dorsal and sagittal planes. The maximum size (mm) of the width of the zone C3 sclerosis (measured from dorsal cortex surface) on transverse plane was graded as mild (<20 mm), moderate (20–30 mm) or severe (>30 mm), and the dorsal and sagittal plane (measured from SCB distally) as mild (<5 mm), moderate (5–10 mm) or severe (>10 mm).

**Data Analysis**

For statistical analysis, limbs in which DR and CT studies had been performed (all within 5 days) were used for analysis. The presence and extent of lesions detected on DR and CT were compared. Agreement between the two modalities for the predefined set of radiographic and CT parameters was determined using Cohen’s unweighted kappa coefficient (K) [28]. K was interpreted as <0 poor agreement; 0.0–0.20 slight agreement; 0.21–0.40 fair agreement; 0.41–0.60 moderate agreement; 0.61–0.80 substantial agreement; and 0.81–1.0 almost perfect agreement [29]. Frequency of uncertainty was calculated as % of ‘unsure’ ratings. Commercial statistical software program (GraphPad Software, Inc., La Jolla, CA, USA) was used for analyses. The presence of a statistically significant difference between the degree of uncertainty on DR (digital radiography) and CT was determined using the Chi-square test (*p* < 0.05).

## 3. Results

### 3.1. Part 1

**Figure 1:**
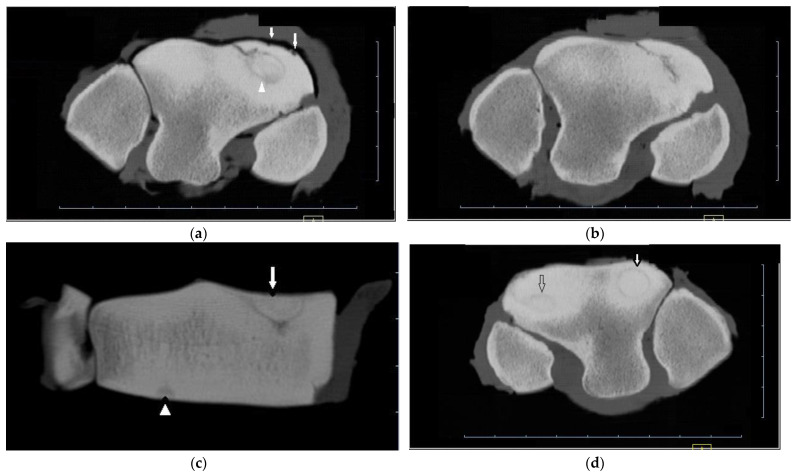
CT images of the distal row of carpal bones of a 5-year-old thoroughbred gelding (post-mortem specimens). (**a**) Transverse CT image of the right carpus just below the proximal articular surface of the third carpal bone (C3), and (**b**) through the distal third of C3. (**c**) Dorsal CT image of the right carpus. There is a circular lucency within the radial facet (arrow head (**a**)) that extends as a Y-shaped lucency into the sclerotic medulla of C3 (arrow (**c**)), and an incomplete frontal fracture extending from this lesion to exit the dorsal cortex of C3 (**a**,**b**). Concurrent lesions include small dorsal cortical lucencies (arrows (**a**)) and a focal lucency within the distal subchondal bone of the intermediate facet (arrow head (**c**)). (**d**) Transverse CT image of the left carpus reveals similar circular lucencies within the radial and intermediate facets (arrows); the lucencies are saucer-shaped on dorsal and sagittal images (not shown).

Horses ranged in age from 2 to 7 yr (mean 4.27 yr) for cases and 1 to 6 yr (mean 3.2 yr) for controls. Film radiographs (XR) were available for the fractured carpus of all 15 horses, and of the contralateral carpus in 6 horses. CT was available of the contralateral carpus of 13 horses. Fracture cases included 2 incomplete and 13 complete Fx, either frontal (*n* = 13) or corner (*n* = 2), and ranged from simple to severely comminuted. Signalment, Fx characteristics and other lesions are described in Supplementary Table S2. XR underestimated or failed to detect bone injury in all cases including comminution (nine horses) or an additional major fissure (one horse, Supplementary Figure S1), bone loss at the proximal articular surface (five horses), partial or full thickness subchondral bone lucency (SCBL) (four horses), medullary lucency in C3 (three horses) and radiocarpal bone (Cr) and or intermediate carpal bone (Ci) lesions (five horses) including osseous fragments (OF) and small, linear, triangular or rounded dorsal cortical lucencies (DCL). Incomplete C3 Fx was only detected on CT in three horses. CT confirmed Fx configuration as corner rather than sagittal in one horse. In another horse, CT identified Y- or saucer-shaped lucency within the RaF and InF of both carpi; an incomplete frontal Fx extended from the RaF lesion (Figure 1). CT confirmed complete Fx in 1 horse when incomplete Fx was diagnosed on XR. Bone injury was detected in the contralateral carpus in eight horses (8/13, 61.5%) including incomplete Fx RaF (two horses), SCBL within C3, Cr and or distal radius (diRd) (six horses) and OF Cr or Ci (three horses); and DCL were present in 2 horses.

Trabecular lucencies of variable size (2–11 mm) appeared on CT as poorly defined, irregular or circular lytic areas usually adjacent to the SCB, and usually surrounded by increased radio-opacity.

As digital radiographs (DR) were not available for these cases, they were excluded from Part 2.

CT findings in the 10 control horses included mild to moderate C3, Cr or Ci sclerosis that was subjectively greater in trained than untrained controls, minor DCL of C3 or Cr (*n* = 2), subtle SCBL within the RaF of C3 (*n* = 1), and second carpal bone (C2), or ulna carpal bone (Cu) lucency with or without associated fragmentation (*n* = 2). The number, location and appearance of vascular channels was noted.

### 3.2. Part 2

A total of 26 horses (24 Thoroughbred, 2 Standardbred), 13 geldings, 10 female and 3 entire males, of mean age 3.61 years (range 1–6 years), were included in the study. As one horse had bilateral C3 Fx, there were a full standard series of radiographs and CT of 27 fractured carpi (14 right and 13 left) used for analysis (Supplementary Table S3). An additional three horses had CT but no DR available of the other carpus.

**Frontal Fractures**

There were 18 frontal plane Fx in 17 horses: 5 were simple, incomplete Fx of the radial facet (RaF) and 13 were complete involving the RaF only (*n* = 8, including 6 uni-articular Fx), intermediate facet (InF) only (*n* = 1, uni-articular) or both facets (*n* = 4,) with nil (*n* = 4), mild (*n* = 7) or moderate (*n* = 3) comminution. One horse with a complete, frontal, RaF Fx had an incomplete, frontal, RaF Fx in the other limb. The 6 uni-articular Fx extended one- to two-thirds the depth of C3 before exiting the dorsal cortex.

**Figure 2:**
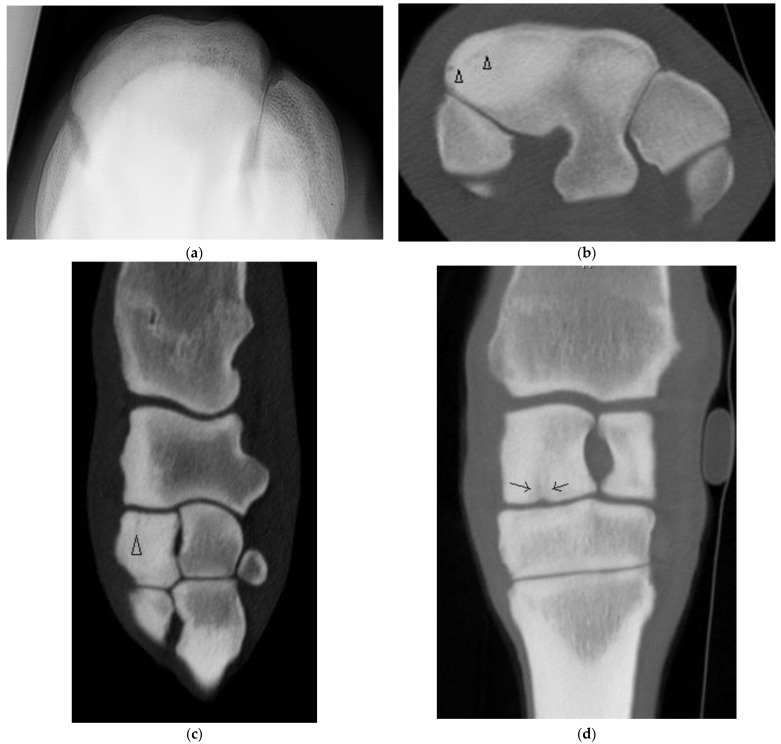
Images of the left carpus of a 3-year-old thoroughbred colt. (**a**) Flexed dorsoproximal-dorsodistal oblique radiographic view of the distal row of carpal bones. There is moderate sclerosis of the radial facet of the third carpal bone (C3). (**b**) Transverse and (**c**) sagittal CT images of the left carpus reveal an incomplete frontal fracture of the radial facet (arrow heads) that was not evident radiographically. (**d**) Within the radiocarpal bone there is a subchondral lucency approximately 4 mm from the dorsal articular margin, vague vertical lucencies that extend into the trabecular bone (arrows) and surrounding sclerosis.

Of the five horses with incomplete, frontal, RaF Fx, one was not evident radiographically and CT was performed due to undiagnosed carpal lameness. On DR of this horse, severe sclerosis of the dorsomedial aspect of the RaF, mild modelling of distal dorsal aspect of Cr and mild entheseous new bone (‘carpitis’) of Cr were reported. CT revealed a subtle, linear, frontal radiolucency within the dorsomedial aspect of the RaF, modelling or osteophytosis (OP) at the C2-C3 articulation and changes within Cr (Figure 2, Supplementary Figure S2). Marked sclerosis was also a feature of the other cases, as were DCL that were more evident on CT than DR (Supplementary Figure S3) and/or mild entheseous new bone Cr, and additional lesions including OF of distal Cr (diCr) and SCBL with vague vertical lucencies that extended into the trabecular bone of diCr not detected on DR (Figure 2).

All horses with frontal Fx had mild to marked additional bone lesions in the Fx carpus (Supplementary Table S3), most commonly SCBL within the RaF or InF of C3, DCL, and SCBL or OF Cr and or Ci.

In addition to sclerosis, all seven horses with complete, bi-articular ‘true slab’ fractures had subchondral and regional bone loss (a ‘trough’) at the proximal articular surface (for an example see Supplementary Figure S4). All had bone fragmentation within the ‘trough’ or comminution that was under-recognized radiographically, and six had palmar debris (recognized on DR in four of six horses).

**Sagittal Fractures**

**Figure 3:**
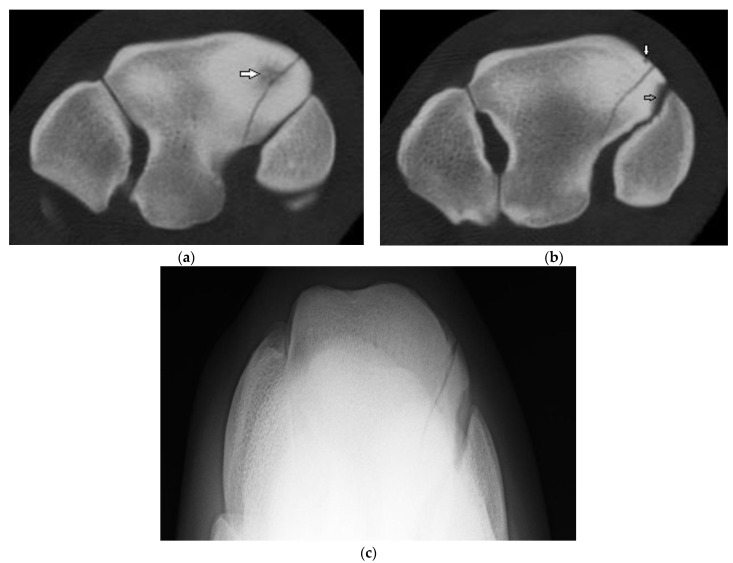
Images of the right carpus of a 3-year-old thoroughbred filly. (**a**) Transverse CT image of the distal row of carpal bones reveal a sagittal slab fracture of the radial facet of the third carpal bone (C3). This slice through proximal C3 reveals a large subchondral lucency within the radial facet that was not evident radiographically. (**b**) A transverse CT image through mid C3 reveals a partial thickness subchondral lucency at the articulation with the second carpal bone (C2) (arrow) and a small dorsal cortical lucency (white arrow). (**c**) Flexed dorsoproximal-dorsodistal oblique radiographic view of the distal row of carpal bones. The sagittal fracture and subchondral lucency at the articulation with C2 is evident but the subchondral lucency mid radial facet is not.

Five horses had a sagittal fracture of the RaF (one incomplete, three complete, and one unsure on DR and CT but determined most likely incomplete); all lacked comminution. In one horse, both observers were unsure whether the incomplete RaF Fx was sagittal or corner on DR; CT confirmed a sagittal Fx. In another horse, both observers were unsure whether the Fx was sagittal or corner and whether the Fx was complete. In both cases, CT confirmed fracture configuration as sagittal and identified additional lesions not identified on radiographs (Supplementary Table S3). Of the three horses with complete sagittal fracture, all had some lysis of the proximal SCB at the fracture margin within the mid RaF (Figure 3) and 2 had additional lesions including DCL and OF not evident radiographically.

**Fractures of Other Configuration**

**Figure 4:**
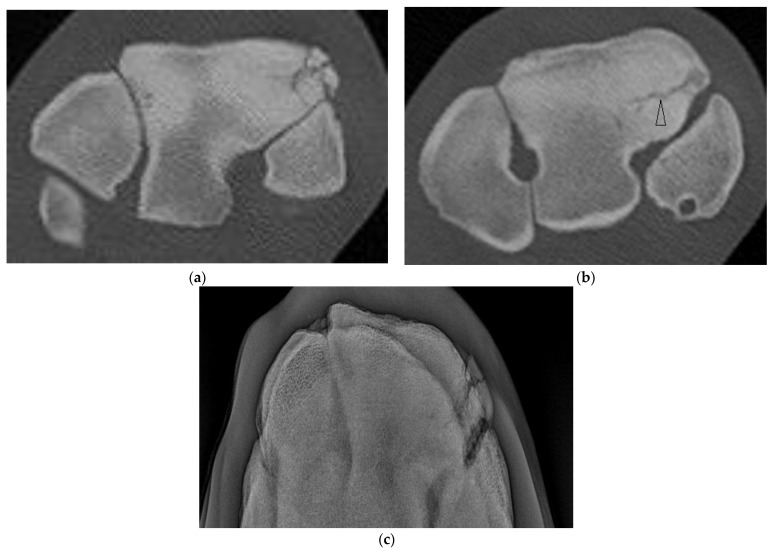
Images of the left carpus of a 6-year-old thoroughbred gelding. (**a**) Transverse CT image through the proximal portion of the distal row of carpal bones. A comminuted corner fracture of the radial facet of the third carpal bone (C3) is evident. (**b**) A transverse CT image further distad reveals a large frontal fissure (arrow head). (**c**) Flexed dorsoproximal-dorsodistal oblique radiographic view of the distal row of carpal bones. The comminuted corner fracture was evident radiographically but the frontal fissure was not.

**Figure 5:**
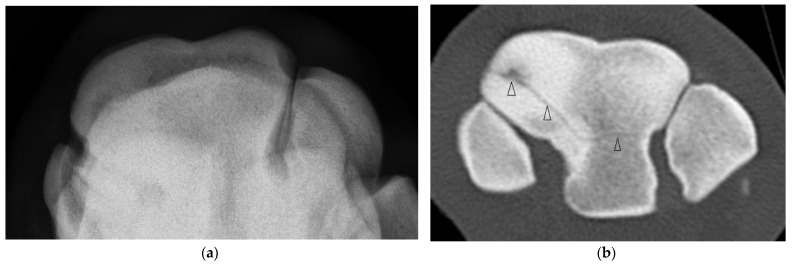
Images of the right carpus of a 3-year-old standardbred filly. (**a**) Flexed dorsoproximal-dorsodistal oblique radiographic view of the distal row of carpal bones. There is a vague sagittal lucency surrounded by sclerosis within the radial facet (RaF) of the third carpal bone (C3). A fracture was suspected but the configuration was unclear. (**b**) Transverse CT image through proximal C3. CT imaging confirmed a complete slab fracture of C3 that is sagittal within the RaF then courses in a frontal plane palmar to the intermediate facet (arrow heads). The fracture passes through a moderate sized subchondral lucency within the RaF.

Four horses had complete fractures of variable configuration with both sagittal and frontal components. Three were corner Fx of the RaF and two of these had substantial additional fissures or fractures detected only on CT (Figure 4). The other horse had a complete Fx of both facets, with orientation that was sagittal in the RaF and frontal palmar to the InF (Figure 5); a configuration not recognized on DR.

**Sclerosis**

All carpi had C3 sclerosis at least mild in severity on CT. Six Fx carpi with mild sclerosis on CT all had non-comminuted (simple) Fx (either incomplete or complete, frontal or corner). All other Fx carpi had moderate sclerosis on CT apart from one with severe sclerosis (a complete sagittal Fx). The width of the zone of C3 sclerosis on transverse plane was mean 22.1 mm (SD 4.2 mm) for Fx carpi and 19.0 mm (SD 3.7 mm) in contralateral limbs and on the dorsal/sagittal planes mean 19.7 mm (SD 3.3 mm) for Fx carpi and 18.8 (SD 3.8 mm) in the contralateral limbs. Of the 38 limbs (27 Fx limbs from 26 horses, and contralateral limb without Fx), C3 sclerosis was assessed as mild, moderate or severe on both DR and CT in 2, 11 and 0 limbs respectively, was scored a higher grade on CT than DR in 1 limb (mod on DR yet mild on CT, *n* = 1; mild on DR yet severe on CT, *n* = 7), and a higher grade on DR than CT in the remaining limbs (mod on DR yet mild on CT, *n* = 4; severe on DR yet mod on CT, *n* = 10).

**Lesions at Other Sites within the Fractured Carpus**

On CT all Fx carpi (27/27, 100%) had Cr sclerosis of the dorsal or only dorsomedial aspect of diCr and 20/27 (74.1%) had one or more additional Cr lesions (Supplementary Table S3) ranging in severity, including OF of the distal Cr (diCr) (*n* = 7) or proximal aspect (PrCr) or Cr (*n* = 1), modelling/osteophytosis of the distal articular margin (*n* = 5) and/or dorsal cortex with entheseous new bone (*n* = 3), DCL (*n* = 6), SCBL 3–5 mm palmar to the dorsal distal articular margin (*n* = 5) or of the mid articular surface (*n* = 2), a vertical fissure through the trabecular bone within the dorsal aspect Cr (*n* = 1), or a comminuted slab fracture (*n* = 1).

Seventeen of 27 (63.0%) Fx carpi had Ci sclerosis (of the dorsomedial aspect of distal Ci) on CT; 10 of these (10/27, 37.0%) with other Ci lesions (Supplementary Table S3) including minor new bone formation on the dorsal distal cortex alone (*n* = 2) or with one or more DCL (*n* = 2), focal SCBL within the sclerotic zone beneath the distal articular surface (*n* = 3), or OF of the proximal (prCi) (*n* = 2) or distal (*n* = 1) aspect (diCi).

Nine of 27 carpi (33.3%) with C3 Fx had at least mild sclerosis of the distal radius (diRd). This was associated with incomplete (*n* = 3), or complete OF (*n* = 2) with new bone formation of the dorsal cortex and DCLs, or with an osseous cyst-like lesion (OCCL) (*n* = 1).

There was bone debris or small osseous fragments within the palmar carpus in five horses and all had complete frontal or corner fracture. Two horses had palmar ulna carpal bone fragments.

**Other Carpus**

Twelve horses had DR and CT of both carpi and were included in the agreement analysis. Both carpi from the horse with bilateral C3 Fx were included in the Fx carpi results. The CT findings in contralateral carpi from the other eleven horses and from an additional three horses that had CT but no DR available of the other carpus had a range of findings including sclerosis (of C3, Cr, Ci, diRd), articular margin, entheseous or dorsal cortical new bone (*n* = 5), DCL (*n* = 6) of C3, Cr, Ci or diRd, OF (*n* = 4) of C3, Cr or Ci, SCBL of the RaF (*n* = 4), InF (*n* = 2), Cr (*n* = 2) or Ci (*n* = 2), vertical lucencies or fissures diCr (*n* = 1) and small, complete, frontal, articular Fx dorsal metacarpus (MCIII) (*n* = 1) (Supplementary Table S3). Overall, 9/15 (60.0%) horses with CT of both carpi had findings in addition to sclerosis, minor DCL and/or minor modelling, osteophyosis or entheseous new bone in the other limb.

**Inter-modality Agreement**

For agreement analysis, 38 limbs were used (27 Fx carpi from 26 horses and 11 contralateral carpi without C3 Fx). Inter-modality agreement results are presented in Suppl. Table S1. Agreement was substantial or almost perfect for recognition of sagittal or frontal Fx only, Fx of InF only, displacement, and presence of bone debris in the palmar carpus, moderate for Fx of RaF only or both facets and C3 DCLs, fair for comminution and the presence of bone loss at the articular surface, and poor to fair for assessing the extent of bone loss. Agreement was substantial for recognition of a complete frontal Fx but for other Fx types agreement for whether the Fx was complete in each plane was poor to slight. Agreement was fair for identifying OF or DCL at other sites (Cr, Ci, diRd), slight for detection of fissures and SCBL and poor for detecting lucencies that involved the trabecular bone.

**Frequency of Uncertainty**

Using consensus results of the two observers for the 27 Fx limbs, greater uncertainty was recorded for DR compared to CT. ‘Unsure’ was recorded for 64/163 (39.3%) DR vs. 6/163 (3.4%) CT observations of whether the Fx was complete in each plane (*p* < 0.00001). In addition, ‘unsure’ was recorded on DR for 7/27 (25.9%) carpi for Fx configuration (frontal, sagittal, or combination frontal and sagittal) with all uncertainty due to challenges classifying corner Fx, for 1/27 (3.7%) for Fx location (RaF, InF or both facets), for 5/27 (18.5%) for presence and grading of comminution, for 15/27 (55.6%) for bone loss at the proximal articular surface and for 3/27 carpi (11.1%) for SCBL while no uncertainty was reported for these parameters on CT. For detection of fissures, ‘unsure’ was recorded for 11/27 (40.7%) DR vs. 6/27 (22.2%) CT (*p* = 0.14). Uncertainty about the presence of vertical lucencies was reported on CT in 3/27 (11.1%) carpi (as these lesions are characteristically subtle) but this parameter was not assessed on DR. In addition, on CT, there was uncertainty about the presence of a very small OF in one carpus and in another case uncertainty differentiating an osteophyte from OF.

## 4. Discussion

For horses with C3 Fx, early and accurate diagnosis of Fx configuration, the extent of bone injury and the presence of concomitant lesions is important when developing a treatment plan [8]. We aimed to evaluate the diagnostic accuracy of conventional radiography by comparison with CT in horses with a range of C3 fracture configurations. This study included horses with C3 Fx configurations representing the range of types previously described [25,26] and in accordance with our hypothesis, CT was superior to radiography for diagnosis of fracture configuration and for evaluating the presence and extent of comminution, bone loss at the proximal articular surface, bone lucencies and injury to other carpal bones which were all identified more often on CT. Some incomplete C3 Fx were only evident on CT. CT was also more useful when determining whether a Fx was complete or not. In addition to greater diagnostic error, or at least incomplete diagnosis with radiography, the degree of uncertainty during image interpretation was greater on radiography than CT.

The deficiencies of radiography are particularly evident when imaging the carpus due to its complex anatomy and superimposition of bones. Although special projections can improve detection of pathology at some sites, for example, sagittal Fx of the RaF [9], the current study confirms that standard views are often not sufficient for consistent detection of pathologic features concurrent with Fx or that may precipitate Fx. Furthermore, although ‘skyline’ views are useful for evaluation of C3, the view can magnify and distort anatomy, and lesions further palmar (>8–10 mm) than the dorsal edge of the proximal row of carpal bones cannot be evaluated due to superimposition. This was an issue with some cases in the current series that included frontal slab width up to 25 mm, frontal or corner Fx with palmar fissure, corner or sagittal Fx that involved the palmar portion of the RaF, or in one case a Fx that extended across C3 palmar to the InF. Diagnostic accuracy with DR can also be limited by inability or failure to obtain projections with the X-ray beam parallel to the Fx plane. It is important to obtain a full series of well-exposed and well-positioned radiographs with additional lesion oriented views as indicated if DR is being heavily relied on for diagnosis. Summation of sclerosis and lucency giving the impression of normal opacity also likely contribute to the inability to detect some lucencies with DR. In contrast, the greater number of lesions and detailed Fx characteristics including the extent of comminution detected with CT can be explained by the higher spatial resolution and cross-sectional nature of this modality. Bone loss and comminution at the proximal articular surface was under-recognised on DR compared to CT. Similarly, comminution of the articular surface was often unrecognized on radiography when compared to CT in horses with lateral condylar Fx of the distal metacarpus [23], another common injury in racehorses.

A high level of uncertainty is inherent to the process of radiographic interpretation [30]. For reasons discussed above, uncertainty in interpretation is prevalent in interpretation of carpal radiographs. Observers were unsure most frequently when assessing radiographs for comminution and bone loss (presence of a ‘trough’ at the proximal articular surface), for determining whether the Fx was complete in each plane and when classifying corner Fx, whilst with CT uncertainty in assessment was recorded in far fewer observations and only in relation to fissures, subtle or small lesions. For detection of fissures, ‘unsure’ was recorded more often for DR vs. CT; yet, the difference was not significant. A thorough knowledge of normal anatomy is necessary to prevent erroneous interpretation with DR and CT. In our opinion studying CT images of the carpus in control horses and bilateral imaging was important for reducing observer uncertainty, and presumably for improving accuracy when interpreting CT.

Accurate diagnosis is unquestionably an advantage for surgical planning [8] and earlier Fx diagnosis is clearly beneficial in racehorses as diagnosis failure could result in catastrophic injury if training continued. For example, in one horse, an incomplete frontal Fx was only detected using CT and in another horse bilateral frontal Fx was detected with CT albeit smaller and incomplete in the other limb. In agreement with other studies [5], fractures most commonly involved the RaF, occurred in a frontal plane, with comminution. Arthroscopy may also help confirm the configuration of articular components of C3 Fx, presence of OF and some other lesions; yet, it is limited to evaluation of surface detects. The low sensitivity of radiography has previously been recognized when comparing DR to arthroscopic findings [5,15,31] and DR to CT in horses with carpal Fx [8]. Reporting how CT findings influenced management and describing arthroscopic findings, surgical management and outcome was beyond the scope of the current study. Yet, we identified structural changes within the fractured and contralateral carpus on CT that in some cases were not apparent on DR or arthroscopy (pers. communication with co-author surgeons), but may warrant surgical debridement such as incomplete or complete OF and SCBL. Furthermore, by accurately determining Fx configuration and whether a Fx was complete or not, decisions regarding whether to lag screw and the positioning of screws was influenced by CT findings (pers. communication with co-author surgeons). In addition to influencing surgical planning, certain CT findings including the extent of comminution and bone loss are considered likely to affect prognosis for future athletic use [32]. Correlation of CT findings, clinical information including arthroscopic findings, and outcome needs to be further explored.

As bilateral carpal injury is common, it is understandable that lesions were commonly identified within the contralateral carpus on CT. We identified an incomplete frontal Fx of the RaF of the contralateral limb in several horses (2 of 15 horses in Part 1 and in 1 of 26 horses in Part 2), and a range of other bone lesions including OF and SCBL that occurred in addition to sclerosis, minor DCL and/or minor modelling, osteophytosis or entheseal new bone in approximately 60% horses. Accurate diagnosis of the presence and extent of bone injury in both limbs is important for surgical planning and prognostication and the current study underscores the value of CT imaging of both carpi in horses with C3 Fx.

Some challenges were encountered when interpreting CT in several horses. Determining whether a non-displaced Fx was complete through all cortices was difficult even with CT in several horses. Similarly, in a small number of horses, differentiation of fissure lines from vascular channels and stress resorption required knowledge of normal appearance and careful evaluation. Differentiation of very small OF and osteophytes can also be challenging if the lesion is smaller than the slice thickness, as observed in a few cases.

Many horses with C3 Fx had bone lesions at other sites within the carpus, mainly diCr, sometimes diCi and occasionally diRd, proximal Ci or Cr, or proximal metacarpus. As the aetiology of most carpal injuries is chronic cyclical (fatigue) overload, our finding of changes consistent with multifocal stress remodelling and bony injury is expected. Although beyond the scope of the current study to relate findings to aetiopathogenesis of carpal injury, CT imaging of horses with C3 Fx, and bilateral imaging, could be useful to improve our understanding of prodromal changes that precede slab Fx, and OF. In the current study, there was subjectively greater sclerosis in trained compared to untrained control horses, OF, SCBL which sometimes extended into the trabecular bone, was observed to occur at sites within the carpus where sclerosis occurred (diCr, di-med Ci, diRd). In addition, all horses with slab Fx had at least mild sclerosis, most had moderate sclerosis on CT and severe sclerosis on DR. Therefore, not all C3 Fx were preceded by marked sclerosis, at least as assessed on CT. It was interesting, although again not unexpected, to observe SCBL that appeared as cresentic, linear, or sometimes Y-shaped lucencies at high load sites in the RaF, distal Cr, distal Ci, resembling palmar/plantar osteochondral lesions (POD) lesions well described in the distal metacarpus/metatarsus of racehorses.

In the current study, apart from one limb, C3 sclerosis was scored at least as severe on DR as on CT, presumably due to summation and superimposition. In comparison, in the metacarpal condyle and proximal phalanx of the fetlock joint in horses, higher scores for bone sclerosis were reported on CT compared to DR [16]. As the presence, degree and extent of sclerosis is more accurately assessed on CT, a 3D imaging modality, than on DR, it seems the scoring system we used for grading sclerosis [27] on radiographs overestimated the severity of change.

Although DR may overestimate sclerosis, it underestimated subchondral (SCBL), trabecular and dorsal cortical lucencies (DCL), particularly subtle lesions, although the clinical significance of these is sometimes uncertain. Nonetheless, detection of focal SCBL within C3 or diCr evident on CT but not DR might be useful to guide surgical probing and debridement during arthroscopy in some horses. Sometimes subtle radiographic changes can be detected in hindsight after viewing CT images, albeit with some uncertainty; however, in the current study, both observers evaluated DR images then CT.

The prognosis for racing following C3 slab Fx is guarded [3,5,31,32,33], although possibly a little better for sagittal Fx according to some [32] but not all studies [33]. In a study that assessed radiographic and arthroscopic findings, severe cartilage damage (defined as full thickness erosion with exposure of SCB) and Fx displacement (with every 1 mm displacement associated with a 25% reduction in the likelihood of racing postoperatively) were associated with less likelihood of thoroughbred and standardbred horses racing post-operatively [33]. In the same study, other factors including dorsopalmar thickness of the frontal fragment, comminution and the presence of OF in the mid carpal joint had no effect; yet, there was a trend for severe C3 lysis (defined as lysis > 5 mm) and reduced likelihood for racing. Loss of SCB and cartilage is important and the extent of bone loss in particular is thought to have a negative impact on prognosis [34]. The current study indicates CT is more accurate than DR in identifying the extent of injury including bone loss and C3 lysis. As noted earlier, our aim was to compare DR and CT assessment of horses with C3 Fx, and describing arthroscopic findings and outcome was beyond the study scope. However, our finding that bone loss at the proximal articular surface is common in many horses with complete frontal and corner Fx in particular, and that additional pathology is common in horses with C3 Fx likely helps explain the poor outcome commonly reported for C3 slab Fx.

Interpretation of images in the current study was facilitated by imaging normal carpi initially and by evaluating limb pairs when available. Prominent widened vascular channels should not be confused with fissure fractures or abnormal dorsal cortical or subchondral bone lucencies. Fissure lines in C3 were evident in several horses with complete frontal or corner but not sagittal Fx as linear lucencies evident on multiple planes, perpendicular to the main fracture on CT images. Interpretation of imaging findings is complicated as certain changes might represent a normal adaptive response of bone to training and imaging a larger number of horses without carpal lameness throughout training would be needed.

This study included cases imaged at different institutions with different CT machines, mostly images under GA, some standing. This was a clinical case series with some cases included retrospectively; DR and CT images were not available of both limbs for all horses. Horses with C3 Fx included in the current study might be biased towards a referral population and those with inconclusive radiographic findings; however, we consider this unlikely to have influenced results as all cases presented for evaluation and treatment of C3 Fx were offered CT and most owners agreed.

Displacement on standing DR was compared to CT images that were obtained under GA in most cases; yet, displacement might be affected by weight bearing. Despite this potential limitation, agreement between the two imaging modalities for displacement was good.

We did not aim to determine agreement between multiple observers as suboptimal agreement between observers is already widely recognized when interpreting radiographs and CT images. Instead, two observers, both experienced in interpreting CT images of the carpus, developed and followed precise criterion definition, interpretation was compared, and differences reviewed and a consensus reached. The observers weren’t blinded to the history and findings on DR, potentially influencing CT image interpretation.

The study has several other limitations including lack of assessing the diagnostic accuracy of CT against a gold standard, either histopathology or microCT. However, CT is considered gold standard for evaluation of bone in the clinical setting. Neither imaging technique we used is able to detect cartilage lesions; however the addition of positive contrast arthrography can assist with identification of cartilage loss on CT examination [35]. Pre-operative standing MRI may be useful in determining Fx morphology and guiding surgical planning [6,36] yet movement can be an issue with standing MRI of the carpus. Arthroscopy is limited to diagnosis of injuries involving the joint surface. It was performed following CT in most horses in the current study, excluding several horses with incomplete sagittal or frontal Fx and several horses considered to have a poor prognosis based on the extent of injury, but it was beyond the scope of the current study to report findings. A further limitation of the current study is that the number of cases with some types of bone pathologies is small. As a result, case numbers are insufficient to determine frequency and prognosis for some lesions.

Most horses had CT imaging immediately before surgery, prolonging anaesthesia time for an estimated 20–30 min, consistent with a recent report [8]. Some of the more recent cases had standing CT which offers the advantage of more complete Fx assessment, prognostication and case selection for repair before general anaesthesia. In addition to reducing GA duration, standing CT offers the advantages of allowing more time to carefully review images before surgery.

## 5. Conclusions

Our study demonstrates the greater sensitivity of CT over DR for detailed evaluation of the carpi in horses with C3 Fx. CT offers clear benefits as it allows assessment of important features of the fracture and concurrent lesions that are likely to affect surgical planning and prognosis, although the clinical relevance and influence of certain CT findings on outcome requires further investigation.

## Figures and Tables

**Table 1:** CT machines and acquisition parameters.

Clinic	GA vs. Standing Sedation	System	kV	mAs	Slice Thickness
Clinic 1	GA	GE Hi Speed FXI	120	150	2.0 mm
Clinic 2	GA	Activion™16	120	150	0.5 mm
Clinic 2	GA	Aquilion Prime SP	135	150	0.5 mm
Clinic 3	GA	Siemens Emotion	110–130	150	0.75 mm
Clinic 3	Standing sedation	Equina Scanner	160	8	1.0 mm

Clinic 1 University of California, Davis; clinic 2 University of Queensland; clinic 3 University of Melbourne.

## Data Availability

Not applicable.

## Supplementary Materials

The following supporting information can be downloaded at: https://www.mdpi.com/article/10.3390/ani13091459/s1, Figure S1: Transverse CT images of the left carpus of a 4 year old Thoroughbred gelding. This horse has a complete displaced corner fracture of the intermediate facet of the left third carpal bone; Figure S2: CT images of the left carpus of a 3 year old Thoroughbred colt with an incomplete frontal fracture of the radial facet of the third carpal bone. Concomitant bone lesions to those shown in Figure 2; Figure S3: CT images of the right carpus of a 5 year old male Thoroughbred with an incomplete frontal fracture; Figure S4: Images of the left carpus of a 3 year old Thoroughbred filly with a complete frontal slab fracture of the third carpal bone (C3). Table S1: Agreement between CT and digital radiography of one or both carpi for 26 horses with third carpal bone fractures; Table S2: Third carpal bone fracture characteristics and other findings based on CT and gross post-mortem dissection and comparison to conventional film radiography (XR) in 15 Thoroughbred racehorses with third carpal bone slab fracture; Table S3: Signalment, fracture characteristics and concurrent findings in the fractured and contralateral carpus on CT imaging of 26 horses with third carpal bone fracture.
