# Supplementary material for: Comparison of Radiography and Computed Tomography for Evaluation of Third Carpal Bone Fractures in Horses
Source: Animals (Basel). 2023 Apr 25;13(9):1459. doi: 10.3390/ani13091459 (PMC10177357; doi:10.3390/ani13091459)
Supplement: Supplementary file 1 [file animals-13-01459-s001.zip › animals-2309418-supplementary.pdf]

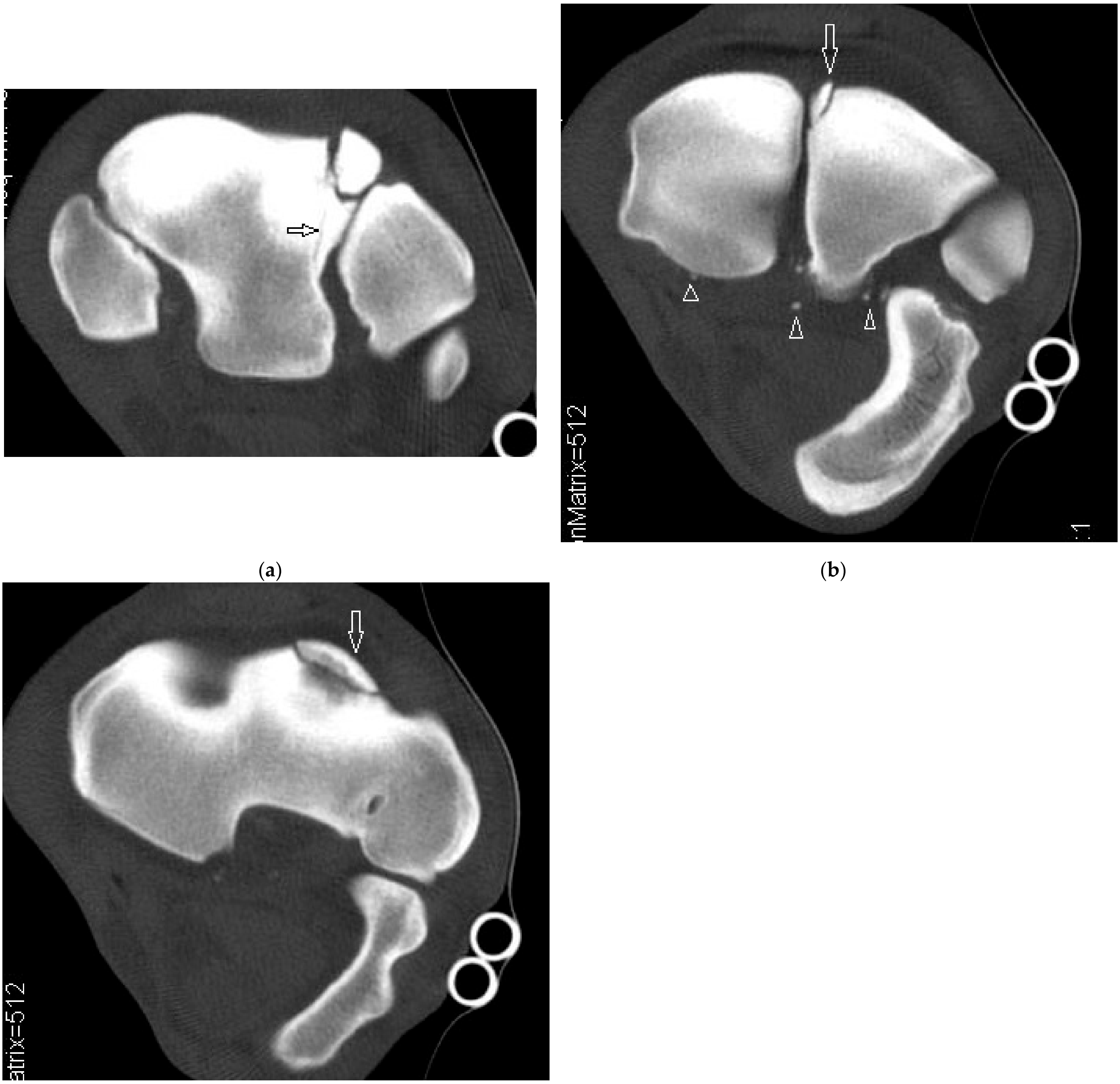

(**a**) (**b**) (c)

Figure S1. Transverse CT images of the left carpus of a 4 year old Thoroughbred gelding. This horse has a complete displaced corner fracture of the intermediate facet of the left third carpal bone. (a) CT identified additional small comminuted fragments within the fracture at the proximal articular surface, and a large fissure extending in a palmar direction (arrow). (b) An osteochondral fragment of the proximal medial corner of the intermediate carpal bone (arrow) and bone debris within the palmar aspect of the joint (arrow heads) is evident. (c) This horse also had and osteochondral fragment with surrounding subchondral bone lysis within the distal radius (arrow).

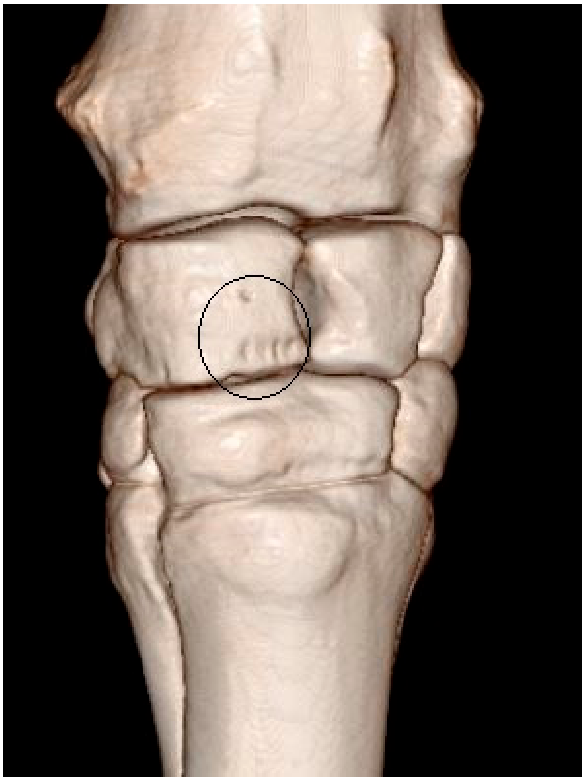

(**a**)

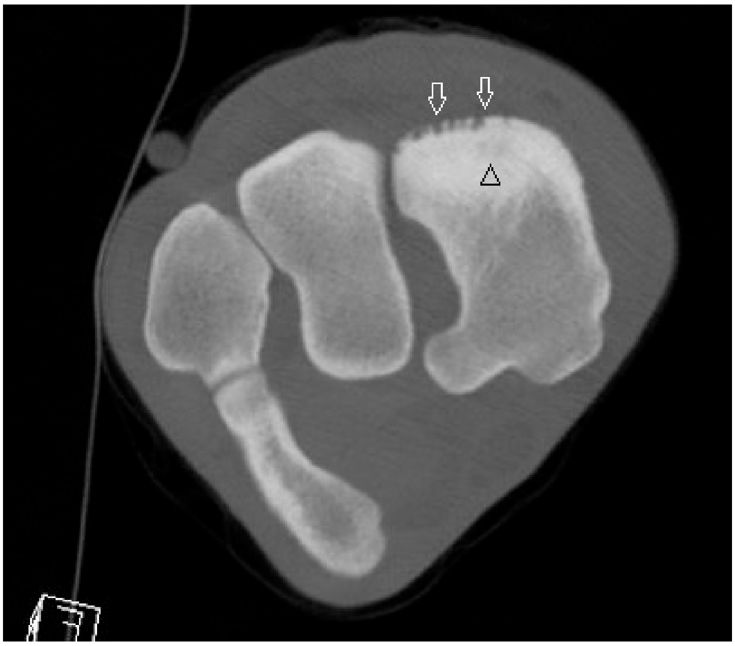

(**b**)

Figure S2. CT images of the left carpus of a 3 year old Thoroughbred colt with an incomplete frontal fracture of the radial facet of the third carpal bone. Concomitant bone lesions to those shown in Figure 2. (a) 3D reconstruction of the carpus showing location of a series of dorsal cortical lucencies (DCL) (circled) within the distal dorsal cortex of the radiocarpal bone (Cr). (b) Transverse CT image of the proximal row of carpal bones at the level of the DCL (arrows) showing a vague lucency (arrow head) that extended from the subchondral bone into the sclerotic medulla. There is marked sclerosis of the dorsal third of the distal Cr. .

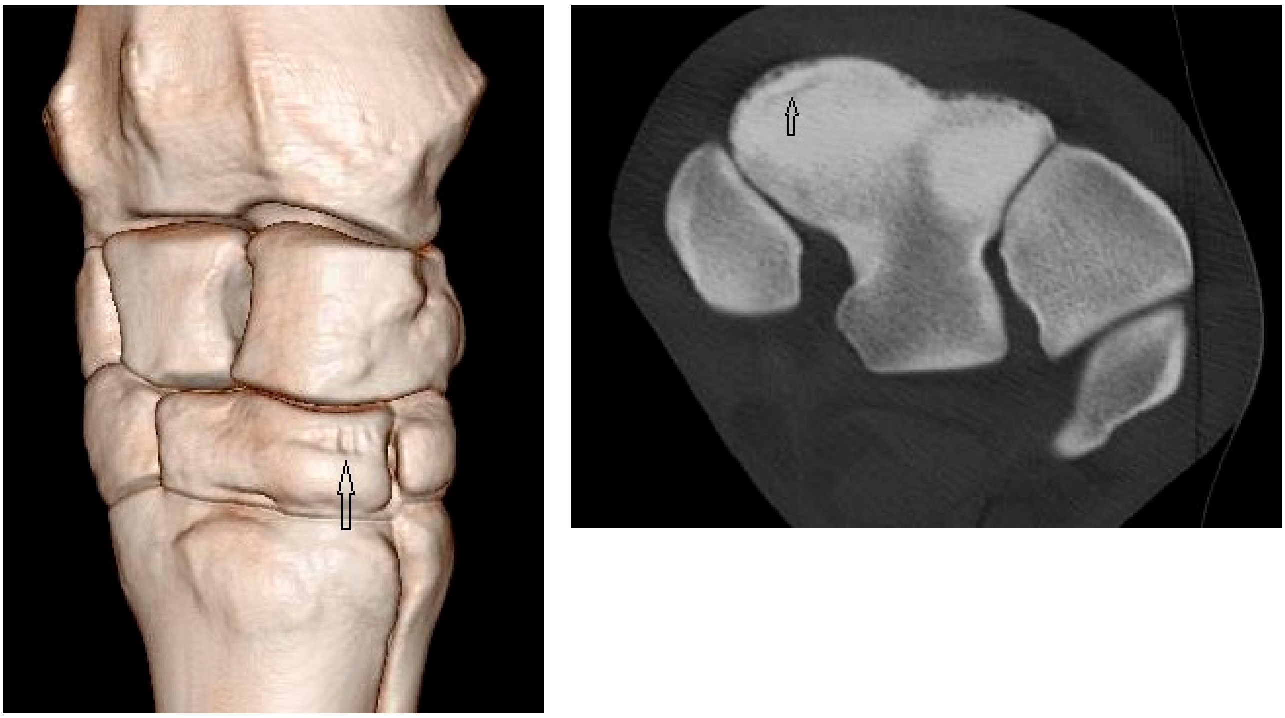

(**a**) (**b**)

Figure S3. CT images of the right carpus of a 5 year old male Thoroughbred with an incomplete frontal fracture. (a) 3D reconstruction of the carpus showing location of a series of dorsal cortical lucencies (DCL) (arrow) within the proximal medial aspect of the third carpal bone (C3). (b) Transverse CT image of the distal row of carpal bones at the level just distal to the proximal subchondral bone showing an incomplete frontal fracture (arrow) and multiple small DCL within the dorsal cortex of C3. There is marked sclerosis of the proximal third of the radial and intermediate facet of C3. .

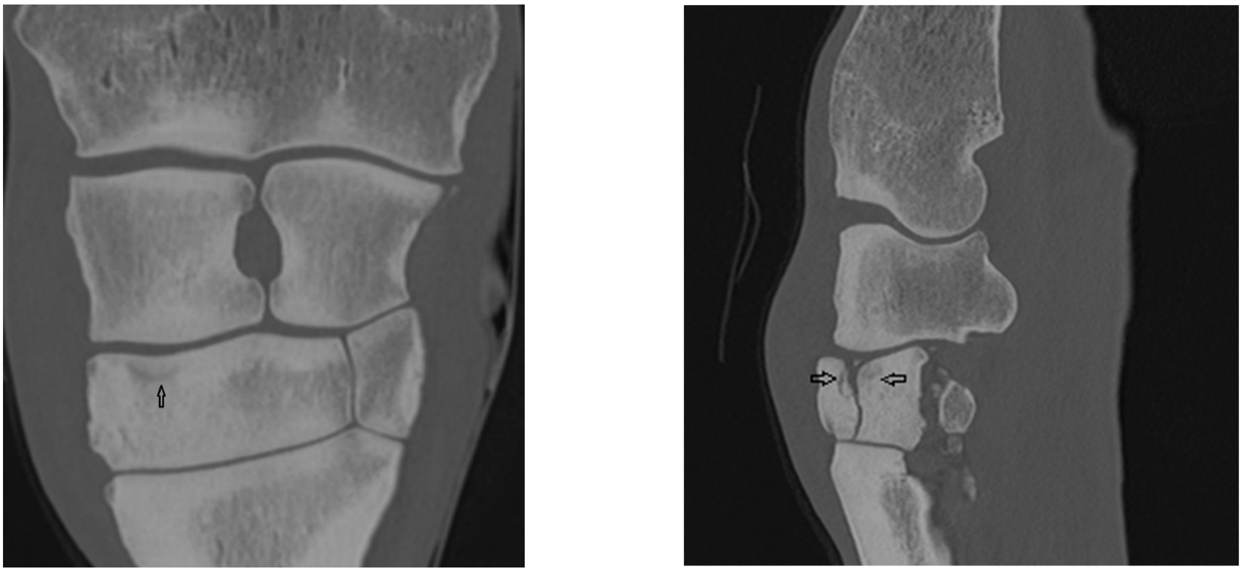

(a) (b)

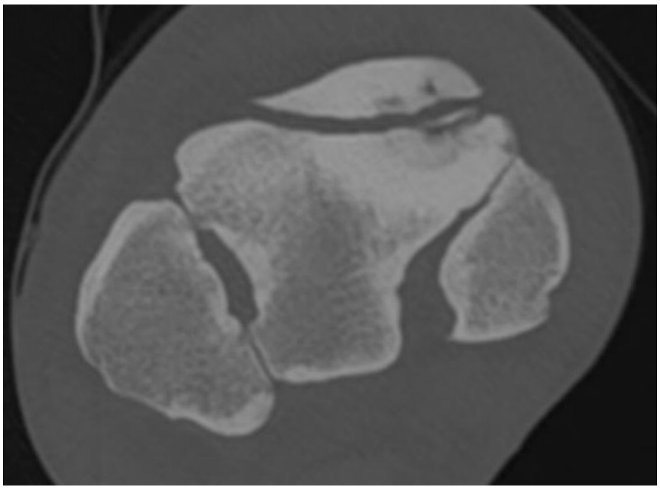

(c)

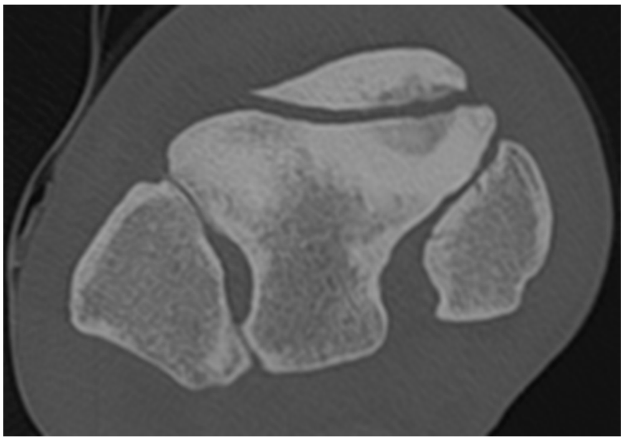

(d)

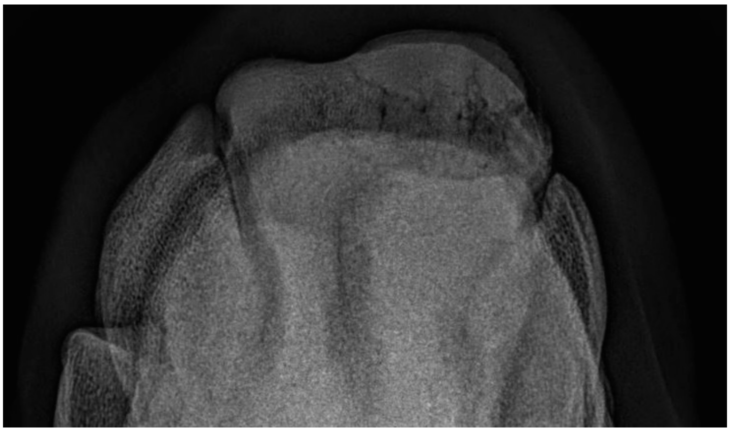

(e)

Figure S4. Images of the left carpus of a 3 year old Thoroughbred filly with a complete frontal slab fracture of the third carpal bone (C3). (a) Dorsal CT image immediately palmar to the fracture showing a crescent shaped radiolucency within the radial facet (RaF) of C3 (arrow). (b) Sagittal CT image through the mid radial facet of C3 showing the complete frontal fracture and surrounding radiolucencies (arrows). There is a V-shaped area of bone loss at the proximal articular surface, and a small fragment within this 'trough'. (c) Transverse CT image of the most proximal aspect of the distal row of carpal bones showing the complete, displaced frontal slab fracture of the RaF and medial aspect of the intermediate facet and a fragment within the fracture at the medial aspect of the RaF. The RaF is sclerotic and there are areas of sclerosis surrounding the fracture. (d) The extent of lysis within the RaF is evident on this transverse CT image slightly more distad. (e) Flexed dorsoproximal-dorsodistal oblique radiographic view of the left distal row of carpal bones showing the frontal slab fracture and areas of lucency within the RaF.

Table S1: Agreement between CT and digital radiography of one or both carpi for 26 horses with third carpal bone fractures. N=number of carpi used for calculation, C3=third carpal bone, Fx=fracture, NC=Not calculable as there were no observations.

Parameter	N	Kappa (SE)	Interpretation
C3 sagittal fracture	38	0.90 (0.09)	Almost perfect
C3 frontal fracture	38	0.74 (0.10)	Substantial
C3 fracture other configuration	38	0.54 (0.20)	Moderate
C3 Fx Radial facet only	38	0.68 (0.12)	Substantial
C3 Fx Intermediate facet only	38	1.0 (0)	Almost perfect
C3 Fx Both facets	38	0.54 (0.18)	Moderate
C3 Fx incomplete sagittal plane cranial not caudal	38	-0.07 (0.033)	Poor
C3 Fx incomplete sagittal plane caudal not cranial	38	NC	
C3 Fx incomplete frontal plane medial not lateral	38	0 (0)	Slight
C3 Fx incomplete frontal plane lateral not medial	38	-0.07 (0.04)	Poor
C3 Fx incomplete proximal to distal - proximal not distal	38	0.13 (0.16)	Slight
C3 Fx incomplete proximal to distal - distal not proximal	38	NC	
C3 Fx complete sagittal plane	38	0 (0)	Slight
C3 Fx complete frontal plane	38	0.72 (0.11)	Substantial
C3 Fx complete proximal to distal	38	0.51 (0.13)	Moderate
C3 Fx comminuted nil (Simple)	38	0.49 (0.15)	Moderate
C3 Fx Comminuted mild	38	0.32 (0.18)	Fair
C3 Fx Comminuted moderate	38	0.72 (0.19)	Substantial
C3 Fx Comminuted severe	38	NC	
Displacement of main C3 bone fragment	37	0.80 (0.11)	Substantial
Main C3 fragment mildly displaced (<5 mm)	37	0.84 (0.11)	Almost perfect
Main C3 fragment moderately displaced (5-10 mm)	37	0.79 (0.21)	Substantial
Main C3 fragment severely displaced (>10 mm)	37	NC	
C3 Fx fissure lines (perpendicular to main fracture line)	38	0.79 (0.21)	Substantial
Bone loss ('trough') at fracture site present	38	0.46 (0.12)	Moderate
Bone loss ('trough') at fracture site small (<5 mm)	38	-0.09 0.05)	Poor
Bone loss ('trough') at fracture site medium (5-10 mm)	38	0.35 (0.20)	Fair
Bone loss ('trough') at fracture site large (>10 mm)	38	-0.04 (0.03)	Poor
Fragment(s) present in proximal fracture line 'trough'	38	0.31 (0.16)	Fair
Focal lysis in proximal subchondral bone plate remote from Fx line present	38	0.30 (0.24)	Fair
Focal lysis in proximal subchondral bone plate remote from Fx line small	38	0 (0)	Slight
Focal lysis in proximal subchondral bone plate remote from Fx line medium	38	1.0 (0)	Almost perfect
Focal lysis in proximal subchondral bone plate remote from Fx line large	38	NC	
C3 bone fragment remote from fracture site	38	0 (0)	Slight
C3 Sclerosis transverse plane mild	38	0.14 (0.13)	Slight
C3 Sclerosis transverse plane moderate	38	0.26 (0.15)	Fair
C3 Sclerosis transverse plane severe	38	-0.03 (0.10)	Poor
C3 Dorsal Cortical Lucencies (DCL)	36	0.51 (0.15)	Moderate
C3 Lucency other than DCL and Fx line	36	0.30 (0.13)	Fair
Lesions in palmar carpus (bone debris or fragments)	38	0.87 (0.12)	Almost perfect
Lesions second carpal bone (C2)	38	0 (0)	Slight
Lesions fourth carpal bone (C4)	38	1.0 (0)	Almost perfect
Lesions radial carpal bone (Cr)	38	0 (0)	Slight

Lesions intermediate carpal bone (Ci)	38	0.25 (0.14)	Fair
Lesions ulnar carpal bone (Cu)	38	0.08 (0.18)	Slight
Lesions distal radius (diRd)	38	0.20 (0.12)	Slight
Soft tissue swelling	36	0.71 (0.12)	Substantial
Osseous fragment(s) (OF) other carpal bone	36	0.34 (0.14)	Fair
Slab fracture other carpal bone	36	1.0 (0)	Almost perfect
Dorsal cortex modelling or enthesis new bone other carpal bone(s)	36	0.43 (0.14)	Moderate
Radial carpal bone (Cr) sclerosis	36	0.11 (0.10)	Slight
Radial carpal bone (Cr) sclerosis without other Cr lesions	36	0.36 (0.17)	Fair
Radial carpal bone (Cr) sclerosis with other Cr lesion(s)	38	0.59 (0.14)	Moderate
Intermediate carpal bone (Ci) sclerosis	36	-0.24 (0.13)	Poor
Intermediate carpal bone (Ci) sclerosis without other Ci lesions	36	0.08 (0.19)	Slight
Intermediate carpal bone sclerosis (Ci) with other Ci lesion(s)	36	0.30 (0.17)	Fair
Distal radius (diRd) sclerosis	36	-0.07 (0.09)	Poor
Distal radius sclerosis without other diRd lesions	36	0 (0)	Slight
Distal radius sclerosis with other diRd lesions	36	0.32 (0.17)	Fair
Dorsal cortex lucencies other carpal bones	36	0.24 (0.16)	Fair
Focal subchondral bone lysis other carpal bone	36	0.07 (0.12)	Slight
Vertical lucency/incomplete fracture other carpal bone	36	-0.04 (0.03)	Poor

Table S2. Third carpal bone fracture characteristics and other findings based on CT and gross post-mortem dissection and comparison to conventional film radiography (XR) in 15 Thoroughbred racehorses with third carpal bone slab fracture. M=male, MC=male castrate, F=female, Fx=fracture, L=left, R=right, C3=third carpal bone, C2=second carpal bone, C4=fourth carpal bone, Cr=radiocarpal bone, Ci=intermediate carpal bone, SCBL=subchondral bone lucency that might extend into trabecular bone, DCL=dorsal cortical lucency, RaF=radial facet, InF=intermediate facet, FT=full thickness, CT=computed tomography, XR=conventional radiography, OF=osseous fragment, med=medial, prox=proximal, compl=complete, POD=palmar osteochondral disease.

Signalment	Fx Limb	Diagnosis based on CT and gross dissection	Lesions identified on CT compared to conventional film radiography (XR)
3 y, M	L	Incompl frontal Fx C3	Incompl frontal Fx, fissures and SCBL RaF (FT Pr SCBL 25% width of RaF) identified on CT not XR
2 y, M	R and L	R comminuted slab Fx C3 with separate OF med corner C3, C4 Fx; L incompl frontal Fx RaF	XR underestimated extent of R C3 comminution and failed to detect incompl Fx L C3
4 y, M	R and L	R comminuted Slab Fx RaF and large OF InF, L C3 incompl frontal Fx	XR underestimated extent of comminution R C3 and failed to detect incompl Fx L C3
3 y, MC	L	Compl frontal slab Fx RaF C3	CT identified large 'trough' bone loss at prox artic surface
4 y, MC	L	Large corner Fx InF C3	CT clarified large corner Fx rather than a sagittal Fx, additional large fissure extending in palmar direction, minor comminution at joint surface, and Ci OF
4 y, MC	R	Compl frontal slab Fx RaF with comminution	CT identified mild prox comminution, additional fissure extending to palmar cortex in prox third of C3, and SCBL RaF in both carpi
5 y, MC	L	Comminuted slab Fx RaF and InF, OF Ci and Cr	Extent of comminution C3 and SCBL Cr detected on CT
5 y, MC	R	Compl frontal slab Fx RaF C3 with additional comminution	Extent of Fx, bone loss and identification of fissure extending in palmar direction. Difficult to obtain good skyline XR due to carpal pain
4 y, F	R	Compl frontal slab Fx RaF C3 with additional comminution	Extent of bone loss and OF in Fx 'trough' and possible fissure lines in palmar direction detected on CT
5 y, MC	L	Compl frontal slab RaF Fx C3 with mild comminution, SCBL distal Ci, OF prox Ci, DCL Cr	Minor additional comminution C3, wide prox bone loss RaF and lesions Cr and Cr detected on CT
4 y, MC	L	Compl nondisplaced frontal slab Fx RaF 3 with focal SCBL InF	CT confirmed slab Fx is complete (appeared incomplete on XR), saucer-shaped SCBL InF, vertical lucencies Cr
6 y, MC	L	Large compl non-displaced corner Fx RaF C3	CT confirmed C3 Fx is complete (unsure on XR) and revealed extent of Cr lesions (SCBL and vertical trabecular lucency, OF and dorsal cortical new bone)
7 y, MC	R	Compl frontal slab Fx RaF C3	Wide prox bone loss RaF, DCLs, lesions Cr and Ci detected on CT
3 y, MC	R	Compl displaced comminuted slab Fx RaF and InF, C2 slab Fx, small SCBL RaF other carpus	CT identified extent of comminution C3, confirming configuration of C2 Fx, trabecular lucency C3, vertical lucencies RaF other carpus
5 y, MC	R	Incompl frontal Fx RaF C3 with saucer-shaped lucency RaF and InF ('POD-like' lesion) both carpi, large OFs diCr other carpus	CT confirmed Fx is incompl and identified saucer-shaped ('POD-like') lesion within prox SCB RaF, lytic defect within distal SCB InF

Table S3. Signalment, fracture characteristics and concurrent findings in the fractured and contralateral carpus on CT imaging of 26 horses with third carpal bone fracture. CT=computed tomography, Fx=fracture, TB=Thoroughbred, SB=Standardbred, C3=third carpal bone, RaF=radial facet of C3, InF=intermediate facet of C3, C2=second carpal bone, Cr=radiocarpal bone, Ci=intermediate carpal bone, Cu=ulna carpal bone, Rd=distal radius, proxMCIII=proximal third metacarpus, L=left, R=right, pr=proximal, di=distal, rmed=medial, lat=lateral, mod=moderate, Sa=sagittal, fr=frontal, incompl=incomplete, uni-artic=uni-articular, bilat=bilateral, SCBL=subchondral bone lucency that might extend into trabecular bone, DCL=dorsal cortical lucency, OF=osseous fragment, OCLL=osseous cyst-like lesion, POD-like=a lucency that is similar in appearance to palmar osteochondral disease of the fetlock. * Indicates changes that were evident on CT but nor DR

Horse	Signalment	Limb	C3 Fracture description				Fissure	Bone loss	Palmar bone debris or fragments	Sclerosis	DCL	Modelling, osteophyte, entheseal new bone	OF	SCBL
			Fx Plane	Complete or Incomplete	Location	Comminution								
**1**	F, 3 y, TB	R	Sa	Complete	RaF	No	No	Medium	No	C3, Cr, Ci	Ci*, Rd*	Rd	Cr(di), Rd	RaF*
	**Comments:** Extent of large SCBL RaF only recognised on CT.													
	**CT contralateral carpus:** Not available													
**2**	G, 2 y, TB	R	Fr	Complete	RaF	Mild*	No	No	Yes	C3, Cr				
	**Comments:** Additional slab Fx of Cr, with extent of comminution Cr best recognised on CT.													
	**CT contralateral carpus:** Not available													
**3**	F, 3 y, TB	L	Fr	Complete	RaF+InF	Mod*	No	Small	Yes	C3, Cr, Ci	Cr			
	**Comments:** Extent of comminution C3 best identified on CT.													
	**CT contralateral carpus:** Not available													
**4**	G, 4 y, TB	L	Fr	Incompl	RaF	No	No	Small	No	C3		Ci*		
	**Comments:** Main benefit of CT was confirming that C3 Fx was incomplete.													
	**CT contralateral carpus:** NA													
**5**	G, 4 y, TB	L	Sag+Fr	Complete	InF	Mild*	Yes*	No	Yes	C3, Cr, Ci	Ci*		Cr (di)?, Ci (pr)*, Rd	Ci (di)*
	**Comments:** CT identified a major additional fissure within C3, palmar bone debris, SCBL Ci and OF Ci not evident on DR.													
	**CT contralateral carpus (lesions other than sclerosis):** OF (nondisplaced) Cr*													
**6**	F, 3 y, TB	R	Fr	Complete, uni-artic	RaF	No	Unsure	Large	No	C3, Cr, Ci			Cr(pr)*, Ci(pr)*, Rd	Cr* (di)
	**Comments:** CT beneficial to identify C3 Fx as complete, OF Cr and Ci, large SCBL RaF and SCBL Cr.													
	**CT contralateral carpus:** Not available													
**7**	G, 6 y , TB	R	Sa	Complete	RaF	No	Unsure	Small	0	C3, Cr, Ci				RaF*
	**Other:** Small OCLL C2.													
	**Comments:** Subtle vertical linear lucencies mid RaF adjacent to Fx line otherwise simple Fx with minimal other bone lesions.													
	**CT contralateral carpus:** NA													

**8**	F, 4 y, TB	R	Fr	Complete, uni-art	RaF	No	RaF*, Cr*	Small	0	C3	Rd*	Ci, Rd	Cr x 2 (di med, di lat*)	
	**Comments:** CT identified sagittal fissure RaF, a second diCr OF, vertical lucency extending into trabecular bone Cr, modelling and DCL Rd all not seen on DR.													
	**CT contralateral carpus:** (CT but no DR). Focal SCBL InF, sclerosis and fissures Cr.													
**9**	G, 5 y, TB	R	Fr	Complete, uni-art	RaF	Mod	Unsure	Medium	0	C3	Cr*, Ci*, Rd*	Cr	Rd (incompl)*	InF (pr)*, InF (di)* and proxMCIII*
	**Comments:** Multiple SCBL, many only evident on CT, incomplete OF Rd evident on CT.													
	**CT contralateral carpus (lesions other than sclerosis):** DCL RaF, Cr, Ci, Rd with incompl OF Rd*, SCBL prox + di InF*, prox MCIII* and prox RaF*, OF C3.													
**10**	G, 3 y, TB	L	Fr	Incompl	RaF	No (unsure on DR)	No	No	0	C3	Cr*, Ci*		Cr (di)?	InF (di)*
	**Other:** POD-like lesion RaF, small palmar axial OF Cu.													
	**Comments:** Extent of incomplete Fx evident on CT.													
	**CT contralateral carpus (lesions other than sclerosis):** Subtle SCBL prCi													
**11**	M, 5 y, TB	R	Fr	Incompl	RaF	No	No	No	0	C3	C3			
	**Comments:** No bone lesions in other carpal bones other than sclerosis.													
	**CT contralateral carpus:** Mild Sclerosis C3 is only finding.													
**12**	M, 3 y, TB	L	Fr	Incompl*	RaF	No	No	No	0	C3	Cr	Cr		Cr (di)
	**Other:** Small incompl Fx/vertical lucency extending into trabecular bone Cr*, Possible OA C2-C3 joint.													
	**Comments:** Marked sclerosis, DCL and SCBL Cr.													
	**CT contralateral carpus:** CT only (no DR). Sclerosis C3, DCL Cr and Rd.													
**13**	F, 4 y, TB	L	Fr	Complete	RaF	Mild	Unsure	No	0	C3	C3	Cr (di OP)*		C3 (RaF prox)
	**Other:** Small OF Cu*													
	**Comments:** Minimal changes other carpal bones.													
	**CT contralateral carpus (lesions other than sclerosis):** Vertical lucencies within trabecular bone and DCL RaF, OA at articulation C2-C3*, multiple small OCLL C2 and MCII, Fx pr MCIII, SCBL and OP Cr.													
**14**	G, 3 y, TB	R	Sa	Incompl?	RaF	No	Unsure	Small	0	C3	Cr*	Cr		Cr*
	**Other:** OCLL distal Rd, separate POD-like SCBL RaF, SCBL or small OCLL distal Rd, small OCF palmar axial Cu.													
	**Comments:** Unsure whether Fx complete or incomplete on CT.													
	**CT contralateral carpus:** CT only (no DR). Severe sclerosis RaF, subtle SCBL mid RaF, minor OP diCr, mild modelling and mild DCL Cr, sclerosis Rd													
**15**	G, 5 y, TB	R	Fr	Complete, uni-artic	RaF	Mild (unsure)	No	Small	1*	C3, Cr, Ci	Cr*			InF (pr)*, Ci (di)*, Cr (pr)*

	**Comments:** SCBL prox InF and 'kissing' SCBL diCi, palmar debris, sclerosis C3, Cr, Ci, DCL Cr. CT detected SCBL InF (bilat) and Ci (bilat) and confirmed OF Cr and Ci within other carpus where only lysis was seen on DR.													
	**CT contralateral carpus:** OF InF C3, SCBL RaF (?), new bone and DCL RaF*, SCBL diCr, OF and SCBL Ci*, DCL and sclerosis Rd*.													
**16**	G, 5 y, TB	R	Fr	Complete	InF	No	No	No	0	C3, Cr, Ci				
	**Comments:** Minimal bone changes in other carpal bones other than some sclerosis.													
	**CT contralateral carpus:** Moderate sclerosis C3, minor DCL RaF.													
**17**	G, 5 y, TB	L	Fr	Complete	RaF	Mild	Yes	Small	1	C3			Cr (di)*	
	**CT contralateral carpus (lesions other than sclerosis):** Incompl Fr Fx RaF, DCL RaF, DCL and entheseal new bone Cr.													
**18**	F, 3 y, TB	R	Fr	Complete	RaF+InF	Mild	No	Small	1*	C3		Cr (OP di)	Cr*, Cu (pa x 2)	Cr (di)*
	**CT contralateral carpus:** Not available													
**19**	F, 3 y, SB	R	Sa+Fr	Complete	RaF+InF	Mild*	No	Small	0	C3				
	**Comments:** CT valuable for determining Fx configuration (as Fx extends palmar to InF) and to confirm Fx is complete.													
	**CT contralateral carpus:** Not available													
**20**	M, 1 y, TB	L	Sa+Fr	Complete, uni-artic	RaF	No	No	No	0	C3		Cr (OP di)		
	**CT contralateral carpus:** Not available													
**21**	F, 2 y, SB	R	Fr	Complete	RaF+InF	Mild	No	Med	Unsure	C3				Cr (di, mid facet)*
	**Comments:** 'Kissing' lesion SCBL Cr, early OF or DCL Rd?*.													
	**CT contralateral carpus (lesions other than sclerosis):** ‘POD-like’ lucency RaF.													
**22**	G, 3 y, TB	L	Sa	Complete	RaF	No	No	No	0	C3				
	**CT contralateral carpus (lesions other than sclerosis):** Not available													
**23**	G, 6 y, TB	L	Sa+Fr	Complete, uni-artic	RaF	Mod*	Yes	Med	0	C3		Cr , C3	Cr (di), Ci(d)	Ci (di)
	**Comments:** Large frontal fissure RaF*, SCBL InF at C4 articulation, multiple OF Cr, Ci, entheseal new bone Cr.													
	**CT contralateral carpus (lesions other than sclerosis):** Multiple OF diCr, OCF diCi, entheseal new bone Cr.													
**24**	G, 4 y, TB	L	Fr	Complete	RaF	Mod*	No	No	0	C3		Cr, Ci		
	**CT contralateral carpus:** Sclerosis and entheseal new bone Cr*, Ci sclerosis*.													
**25**	F, 2 y, TB	L	Sa	Incompl	RaF	No	No	Small	0	C3		Cr	Cr (di)	
	**CT contralateral carpus:** Not available but also had SCBL C3 debrided during arthroscopy.													
**26**	F, 3 y, TB	L	Fr	Complete	RaF+InF	Mild*	No	Large	0	C3, Cr, Ci, Rd			Rd (small incompl)*	C3 (Large Raf)
	**Comments:** Large SCBL central RaF underestimated on DR.													
	**CT contralateral carpus:** Sclerosis C3, Cr and Ci, and subtle vertical lucencies extending into trabecular bone mid RaF*.													
